# Infant Mortality: Groping for a Safe Basis of Indubitable Fact

**Published:** 1917-03-17

**Authors:** 


### INFANT MORTALITY.

### Groping for a Safe Basis of Indubitable Fact.

The subject of infant mortality was recently brought before the Statistical Society by Dr. John Brownlee, Director of Statistics to the Medical Research Committee, in a paper of very great interest, though, of course, a large part of it was occupied by technical statistical matter which it would be out of place to examine here. The following is a summary of the non-mathematical portion.

In the last ten years the problem of the waste of child-life has come more and more prominently to the front. At the present time administrative measures of many kinds are being taken with a view to save infantile lives, and as to the expediency of saving the lives of infants there are three points of view.

First, there are many who hold that every infant life saved adds another potentially fit member to the working power of the State. This opinion is, of course, intensified by the heavy demand that the war is making upon what is called the man-power, to which must be added the woman-power, of the country, and is come so much to the fore that it is now a fashionable fad for ladies, who, as Dr. Brownlee sarcastically says, have either no children themselves, or have had their children brought up by strangers in their houses, to start societies with a view to extend some specious formula of quackery. We may well pray to be saved from the ignorant, well-meaning enthusiast.

Second, there is the view, of which Professor Karl Pearson is the chief exponent, that the saving of infant lives can lead only to an increase in the number of unfit persons who must be a burden on the State.

### The Best Sort of Eugenics.

The third view is that, though the universal saving of infants to live to adult life, supposing it were possible, would undoubtedly add to the number of unfit State-supported persons, yet, given moderately healthy conditions of life, the number of infants that it is practicable to save would include a very much smaller proportion of the unfit than is assumed by Professor Pearson; for that most of the unfitness that exists is due to environmental conditions in early life, and that if these conditions were ameliorated, not only would infant lives be saved, but also many that now survive as unfit would never become unfit. It is to this third party that Dr. Brownlee gives his adhesion, and he has for associates many of the most able medical officers of health in the country, whose opinion is presumably more likely to be correct than that formed solely in his statistical laboratory by Prof. Pearson, unless, indeed, his statistical methods are founded on facts selected with impartial care, and are conducted with unimpeachable accuracy. Dr. Brownlee is of opinion that the data on which Professor Pearson founds his statistics—that is to say, the numbers that he takes as the starting-point of his mathematical manipulations—are not sound, and that the mathematical processes themselves are not to be depended upon.

Into these statistical speculations—for they are, as Dr. Brownlee admits and asserts, highly speculative—want of mathematical dexterity will not allow us to follow him, nor is it likely that we should interest our readers if we could; but certain facts emerge which are of great interest even to those who have no mathematical proficiency. In the first place, we find that such a term as "the infant mortality rate" has two or three distinct meanings. It may mean the number of children who die at an age of less than one year, and this would seem to the non-statistical mind the only correct meaning. But it may mean the number of children who in any one year die at an age less than one, compared with the number of infants born that year. This will, of course, give a very different figure. Again, it is rather surprising to hear that there is at present no sound method of measuring the mortality of children less than five years of age. It is, says Dr. Brownlee, an undiscovered country from whose bourne if any traveller has returned it is quite certain that he has brought back no exact information.

### The Limitations of Statistics.

Again, it is surprising to find that the earlier the parents of a slum child die, the better chance that child has of survival, for it will then be taken out of the slum at an earlier age by the ministering guardian, and placed in the country in healthy surroundings. The death-rate of children under five is said by Dr. Brownlee to have been steadily decreasing for many years, but how this result has been arrived at in the absence of any sound method of measuring this mortality he does not tell us. He tells us, however, that there is no doubt that epidemic diseases occur with a biennial periodicity. It is something in such a world of uncertainty to be able to exclude doubt of anything, but this is not the only matter of which Dr. Brownlee is certain. He is certain that phthisis is not a single disease, but a mixture of two, or probably three, diseases, and that one of these—whose greatest mortality is between the ages of twenty and twenty-five—is more prevalent in Scotland and is more deadly, while the other is more frequent in London, and does not produce its maximum of mortality till the age of about forty. In spite of the fact that Dr. Brownlee is able to arrive by statistical methods at these certainties, the general impression left upon an outsider by a perusal of his paper is that statistical methods are utterly untrustworthy, and that it would be most unsafe to act upon any conclusion reached by their means. Dr. Brownlee satisfactorily demolishes the conclusions that Professor Pearson has founded on his methods, and we have little doubt that Professor Pearson could with equal destructiveness demolish Dr. Brownlee's.

